# The diagnosis and management of pheochromocytoma and paraganglioma during pregnancy

**DOI:** 10.1007/s11154-022-09773-2

**Published:** 2023-01-13

**Authors:** Roderick J. Clifton-Bligh

**Affiliations:** 1 University of Sydney, Sydney, NSW Australia; 2 Department of Endocrinology, Royal North Shore Hospital, St Leonards, NSW 2065 Australia

**Keywords:** Pheochromocytoma, Paraganglioma, Pregnancy, Adrenal medulla, Catecholamines

## Abstract

Diagnosis of pheochromocytoma or paraganglioma (PPGL) in pregnancy has been associated historically with high rates of materno-fetal morbidity and mortality. Recent evidence suggests outcomes are improved by recognition of PPGL before or during pregnancy and appropriate medical management with alpha-blockade. Whether antepartum surgery (before the third trimester) is required remains controversial and open to case-based merits. Women with PPGL in pregnancy are more commonly delivered by Caesarean section, although vaginal delivery appears to be safe in selected cases. At least some PPGLs express the luteinizing hormone/chorionic gonadotropin receptor (LHCGR) which may explain their dramatic manifestation in pregnancy. PPGLs in pregnancy are often associated with heritable syndromes, and genetic counselling and testing should be offered routinely in this setting. Since optimal outcomes are only achieved by early recognition of PPGL in (or ideally before) pregnancy, it is incumbent for clinicians to be aware of this diagnosis in a pregnant woman with hypertension occurring before 20 weeks’ gestation, and acute and/or refractory hypertension particularly if paroxysmal and accompanied by sweating, palpitations and/or headaches. All women with a past history of PPGL and/or heritable PPGL syndrome should be carefully assessed for the presence of residual or recurrent disease before considering pregnancy.

## Introduction

Pheochromocytomas and paragangliomas (PPGLs) are tumors of the adrenal medulla and paraganglia, respectively, that often secrete catecholamines leading to hypertensive crises which may be fatal [1]. Although PPGLs occur rarely in pregnancy, if unrecognised they can be associated with a high risk of maternal and/or fetal mortality. Nevertheless, improvements in medical, obstetric and anaesthetic care over recent decades has been accompanied by a steady fall in these bleak odds from around 50% in 1971 [2] to < 10% in recent times [3–5]. The major challenge remains diagnosis of PPGL as a rare but possibly life-threatening cause of hypertension in pregnancy; when misdiagnosed as pre-eclampsia, maternal mortality can still be as high as 15% [5]. Conversely, diagnosis and medical treatment before delivery is today generally associated with good materno-fetal outcomes [4, 5].

This review will highlight recent seminal work on PPGL in pregnancy, focussing on two recent systematic reviews [4, 5], one of which included 197 additional cases from an international registry-based study [4].

## Normal catecholamine physiology in pregnancy

In healthy pregnant women, plasma and urinary catecholamines do not appear to vary from pre-pregnant normal ranges [6, 7]. Even in pre-eclampsia, maternal plasma catecholamines levels may only be mildly increased [6]. Catecholamines may rise at the time of parturition [8], and the tocolytic (contraction suppressing) effect of beta-adrenergic signalling is sometimes targeted for therapeutic purposes in threatened pre-term labour [9]. Increased placenta clearance protects the fetus from cardiovascular and metabolic effects of stress-mediated catecholamine release [10]. Recent work identifies the fetal adrenal medulla as being responsive to hypoxia [11] and a surge in fetal catecholamines at the time of birth plays an important role in adaptation to post-natal life [12].

## Incidence and clinical features of PPGL during pregnancy

PPGL in pregnancy is rare. Estimates on the incidence of PPGLs in pregnancy vary widely between 1/15,000 and 1/300,000 pregnancies [13, 14]; since the prevalence of hypertension in pregnancy is 3–5% [15], PPGL may therefore be expected to be associated with around 1/450–15,000 of all hypertensive pregnancies. Most PPGLs in pregnancy appear to occur in primiparas [4].

**Fig. 1:**
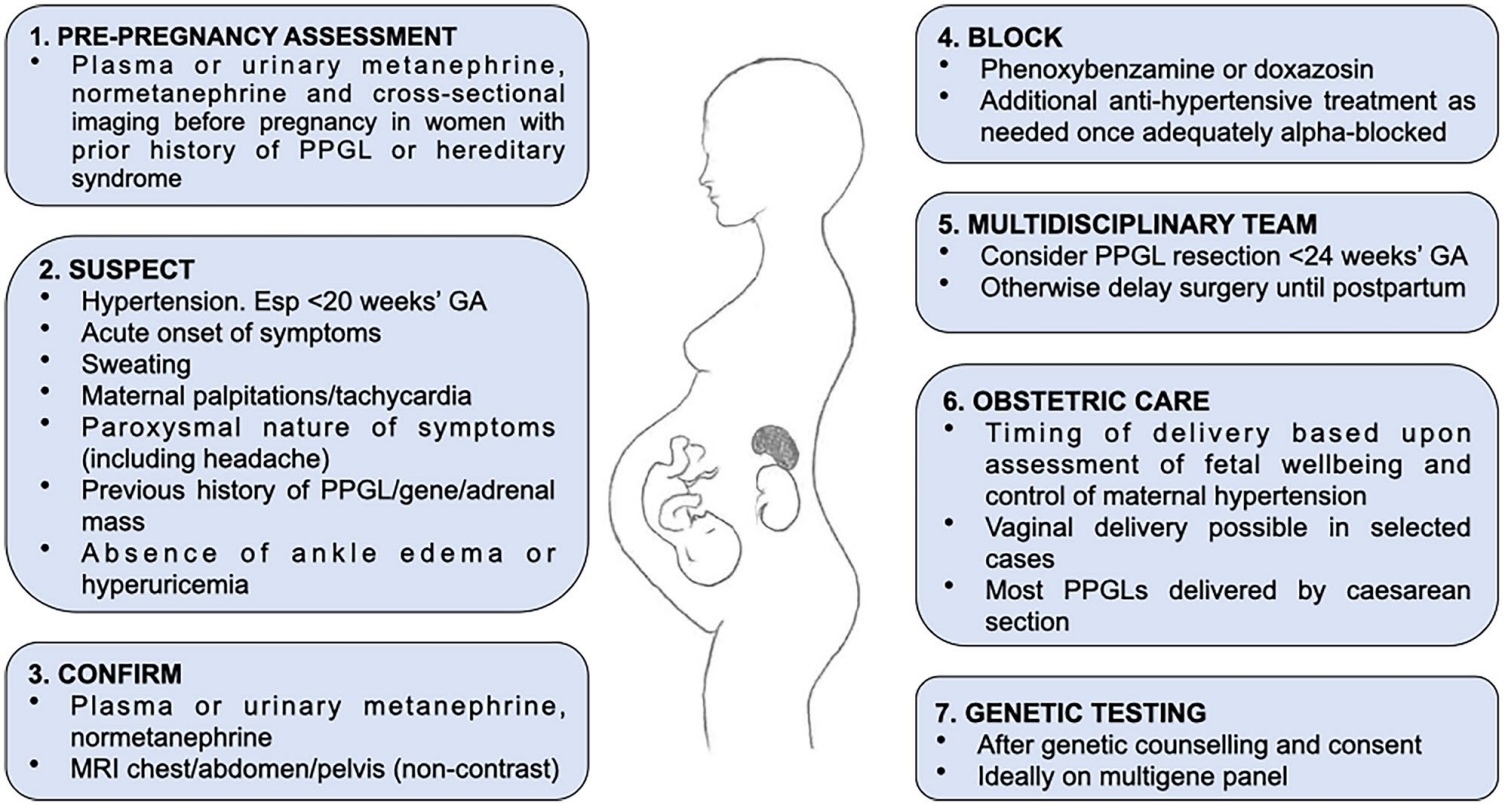
Stages in managing PPGL in pregnancy

The clinical features of PPGL are similar in pregnancy to those classically reported outside of pregnancy, including paroxysms of sweating, palpitations/tachycardia and headache (Fig. 1). Hypertension is common (Table 1) and is the presenting feature in around 20% PPGLs in pregnancy [5]. Hypertension associated with PPGLs may be sustained, paroxysmal or both [6]. The natural reluctance to perform imaging during pregnancy (other than obstetric ultrasounds) would bias against incidental discovery of PPGLs, now an otherwise common presentation of PPGLs outside of pregnancy [16]. Around 30% PPGLs in pregnancy are still not diagnosed until the post-partum period (Table 1) [4, 5]. Of PPGLs diagnosed during pregnancy, approximately half are diagnosed in the third trimester (Table 1) [4, 5].

**Table 1:** Summary of clinical, biochemical and pathological findings of PPGL in pregnancy from two systematic reviews [4, 5]

		Bancos et al. [4]	Langton et al. [5]
Method of review		Combined international retrospective multi-center study based on the newly founded International-Pheochromocytoma-and-Pregnancy-Registry of patients with PPGL and pregnancy occurring between 1980 and 2019 and a systematic review of literature conducted on studies published between January 1st, 2005 to December 27th, 2019. Case reports/series with < 5 patients excluded	Case reports on PPGL in pregnancy published between 1 January 1988 and 30 June 2019 in English, German, Dutch or French
Total (patients/pregnancies)		232/249	200/204
Age, y		29 (range 15, 46)	29 (IQR 25–34)
Weeks of gestation (antepartum diagnosis)		24 (range 2, 38)	25.6 (IQR 18, 34)
Symptomatic during pregnancy		83%	90%
Hypertension		77%	72%
History of PPGL, mutation carriers, adrenal mass		NR	12%
Diagnosis of PPGL	pre-pregnancy	15%	
	during pregnancy	54%	71%
	after pregnancy	31%	22%
	post-mortem		7%
Pathology	PC	61%	70%
	Bilateral PC	8%	9%
	PGL	18%	18%
	Multiple PGL	5%	3%
	Metastatic PPGL	9%	NR
Alpha-blockade		NR	67%
PPGL surgery	During pregnancy	18%	22%
	After pregnancy	70%	77%
	no surgery ator	12%	
Delivery	Vaginal	31%	22%
	Caesarean section	59%	76%
Catecholamine secretion	Total	95%	NR
	Norepinephrine	44%	NR
	Epinephrine	39%	NR
	Dopamine	1%	NR
Family history		25%	NR
Positive genetic test*		66%	23%
Maternal mortality	Total	1.3%	9%
	Antepartum	0%	0.7%
	Postpartum	4%	29%
Fetal mortality	Total	7%	14%
	Antenatal	9%	10%
	Postnatal	13%	25%

*PPGL* pheochromocytoma and paraganglioma, *PC* pheochromocytoma, *PGL* paraganglioma, *ator* at time of report

*genetic testing had been performed in 144 patients in [4]; the number of patients who had genetic testing was not reported in [5]

An important barrier to the diagnosis of PPGL in pregnancy is distinguishing it from much more common diagnoses of gestational hypertension and/or pre-eclampsia [15]. Clues to a diagnosis of PPGL rather than pre-eclampsia include new-onset hypertension before 20 weeks’ gestation [15]; paroxysmal hypertension; and orthostatic hypotension, which can occur in PPGL but almost never in gestational hypertension [6]. Conversely, PPGL-related hypertension is generally not associated with ankle edema or an increased plasma uric acid level, which would otherwise be common clinical signs in gestational hypertension or preeclampsia [6].

## Diagnosis of PPGL in pregnancy

Clinical suspicion for PPGL in pregnancy should be followed by prompt biochemical testing in the same manner as for nonpregnant patients, although it should be noted normal ranges have not been clearly established for pregnant subjects [6]. Fasting supine plasma normetanephrine, normetanephrine and 3-methoxytyramine are now established as most accurate for diagnosis of PPGL in non-pregnant subjects [17], although 24 h urinary fractionated metanephrines and catecholamines are still considered a valid diagnostic approach [18]. A careful medication history is required to avoid false-positive results from pharmacodynamic interference, e.g. from tricyclic antidepressants. Liquid chromatography/tandem mass spectrometry assays are not subject to analytical interference from drugs such as methyldopa or labetalol that may otherwise confound liquid chromatography/electrochemical detection assays [6].

The median size of PPGLs in pregnancy is > 5 cm [4]; at this size, metanephrine and/or normetanephrine measurements are typically elevated at least twice the upper limit of normal [17, 19]. For tumors < 2 cm, metanephrine and/or normetanephrine may be just above the reference range [19]. Evidence suggests the effect of PPGLs on pregnancy depend upon the magnitude of catecholamine secretion: catecholamines or metanephrines > tenfold above the upper limit of normal are associated with adverse materno-fetal outcomes [4].

Following biochemical confirmation of PPGL, imaging is best performed by MRI in pregnancy, without gadolinium [6, 18]. Functional imaging with MIBG or PET is contraindicated in pregnancy [6, 18] but may be appropriate in the post-partum period particularly if bilateral, multifocal or metastatic disease is suspected [3]. Unilateral pheochromocytomas account for 60–70% of PPGLs in pregnancy; bilateral pheochromocytomas for 8%; solitary paragangliomas for 18% and multiple paragangliomas for 2.5–5% (Table 1) [4, 5]. Diagnosis of metastatic PPGL is rare during pregnancy; carefully selected patients with pre-existing metastatic PPGLs may have successful pregnancies with appropriate medical and obstetric care [4].

## Complications of PPGL in pregnancy

Undiagnosed PPGL increases the risk of maternal and fetal morbidity and mortality. Even with improved outcomes reflected in the two most recent systematic reviews, maternal mortality was 4–9% and fetal mortality 7–14% (Table 1) [4, 5]. Both studies found maternal and fetal mortality were unequivocally higher with PPGL diagnosed postpartum than antepartum, including deaths before delivery could occur; conversely, diagnosis of PPGL antepartum was associated with very low risk of maternal mortality (< 1%) and lower risk of fetal mortality (Table 1) [4, 5]. Notably, in most cases of maternal death, a history of PPGL symptoms was evident antemortem [5]. Severe maternal morbidity occurs in up to 7% pregnancies complicated by PPGL, including cardiac failure and neurological events with persistent sequelae [4]. Cardiovascular events are more likely in the peripartum period [6]. Fetal morbidity includes intrauterine growth restriction (IUGR) from sustained maternal hypertension [4–6].

In Bancos et al., factors associated with adverse outcomes, other than unrecognised diagnosis of PPGL during pregnancy and markedly elevated catecholamines, included PPGLs located in the abdomen or pelvis (as opposed to thoracic or head/neck PGLs) and lack of alpha-blockade (see below) [4]. Interestingly, neither maternal age nor tumor size were associated with adverse outcomes in that study [4].

## Management of PPGL in pregnancy

Once diagnosed, pregnant women with PPGL should ideally be managed in expert centres with multidisciplinary access to high-level obstetric and neonatal care, endocrinologists and anaesthesiologists experienced in the management of catecholaminergic crises [20]. There are four components to managing PPGL in pregnancy: alpha-blockade, PPGL surgery, obstetric care and genetic testing for hereditary PPGL syndromes (Fig. 1).

### Medical

Adverse consequences to mother and fetus from PPGL appear to be primarily mediated by markedly elevated catecholamines [4, 5]. Adequate alpha-adrenoceptor blockade for at least two weeks has been associated with improved outcomes [4, 21]. The two most used alpha-adrenoceptor blockers are phenoxybenzamine (non-selective) and doxazosin (alpha-1 selective), without either being clearly superior to the other to achieve normotension in pregnancy [4, 6]. Phenoxybenzamine might be preferred for patients with larger tumors and/or higher catecholamine levels [18]. However, phenoxybenzamine does cross the placenta and may cause hypotension and respiratory depression in the newborn [22, 23]. Although doxazosin also crosses the placenta, there are no reports of adverse events in newborns of mothers treated with this agent [24]. Breastfeeding appears to be safe on either agent [6].

Beta-blocking agents can be added in to control tachycardia after adequate alpha-blockade has been achieved (usually at least a week); the addition of calcium channel blockers may also be needed in some cases to control resistant hypertension [18]. Achieving adequate control of hypertension in pregnancy is crucial for maintaining uteroplacental circulation and avoiding IUGR or fetal demise [25].

Medications which should be avoided in a pregnancy associated with PPGL include metoclopramide, steroids and sympathomimetics all of which may trigger sudden catastrophic catecholaminergic release and hypertensive crisis [26].

### PPGL surgery

Diagnosis of PPGL is usually regarded an indication for surgical resection, after appropriate alpha-blockade [1]. Timing of such surgery remains controversial for PPGLs diagnosed in pregnancy. Surgery is often advocated when PPGL is diagnosed before 24 weeks’ gestation; when PPGL is not diagnosed until the last trimester, term delivery should generally occur before tumor resection [6]. In both recent systematic reviews, more than 2/3 cases had PPGL resection after pregnancy (Table 1) [4, 5], and neither found a significant association between antepartum resection and improved materno-fetal outcomes [4, 5].

Langton et al. noted twice as many women carried pregnancies to term when tumors were resected antepartum (81%) compared with postpartum (41%); and fetal distress (measured by Apgar score) was higher when the tumor was not removed before delivery [5]. Overall, it is generally accepted timing of surgical resection should be made on a case-by-case basis [4, 5].

### Obstetric care

In recent reviews, more than 2/3 of pregnancies associated with PPGLs were delivered by Caesarean section (Table 1) [4, 5]. There is increasing evidence for successful vaginal delivery with adequate alpha-blockade in selected cases [4, 5, 27]; an elective epidural and passive second stage may be prudent to minimise the risk of tumor stimulation from raised intra-abdominal pressure during maternal pushing [27]. Caesarean section is still preferred for women with larger tumors, particularly within the abdomen and pelvis and with higher catecholamine levels [3, 4]. Timing of delivery is usually adjudicated on fetal wellbeing and control of maternal hypertension; the presence of IUGR, decreased fetal movements, fetal cardiac decelerations and/or labile maternal blood pressure are considered indications for early delivery [3].

### Genetics

PPGLs are the most highly heritable of human tumors with 30–40% cases associated with germline pathogenic variants (GPV) in one of up to 16 genes [1]. Bancos et al. found that of patients with PPGL in pregnancy who had genetic testing, 66% had a germline pathogenic variant (GPV) for hereditary PPGL (Table 1), including 19% in *SDHB,* 13% in *VHL* and 19% in *RET* [4]. This distribution of GPVs is broadly comparable to that in PPGL occurring outside of pregnancy; for instance, in a recent large retrospective cohort of PPGLs (n = 1727) using multigene panel testing, the most common genetic association was *SDHB* (11%), followed by *SDHD* (5.8%), *SDHA* (2.8%), *VHL* (2.1%), *SDHC* (1.8%), *RET* (1%) and *MAX* (1%) [28]. The higher rate of GPVs in general, and particularly *RET* mutations in PPGL in pregnancy might be explained by several factors. It is known that younger subjects with PPGL are more likely to have a GPV; in pediatric/young adult series, up to 70% of patients have a GPV in either *SDHB* or *VHL* [29]. Additionally, at least some of these *RET* affected individuals had a known diagnosis of Multiple Endocrine Neoplasia type 2 (MEN2) prior to pregnancy. Whether *RET* associated PPGLs are more likely to declare during pregnancy needs further study; certainly, several case reports attest to the often-dramatic presentation of MEN2-related pheochromocytoma in pregnancy [30–35].

From the high rate of hereditary diagnosis, it naturally follows that all patients with PPGLs in pregnancy should be offered genetic counselling and testing, ideally on a multigene panel containing at least *RET*, *SDHB*, *SDHC*, *SDHD, SDHA, VHL*, *TMEM127* and *MAX*. A positive finding has important ramifications for the patient, her child and other first-degree relatives; gene-specific recommendations for follow-up are discussed elsewhere [36].

It also follows that all female patients with a known hereditary PPGL syndrome should be carefully assessed for tumors using biochemical testing and cross-sectional imaging at the time of pregnancy planning [4, 18]. Pre-implantation genetic diagnosis may also be relevant to discuss with women at this time [37].

## Expression of LHCGR in pheochromocytomas

Although mechanical factors such as tumor compression by the gravid uterus and/or labor are usually invoked to explain marked catecholamine release from PPGLs in pregnancy [4], a new explanation for this phenomenon has been reported by finding increased expression of the luteinizing hormone/chorionic gonadotropin receptor (LHCGR) in at least some PPGLs [38]. The authors reported a case of pheochromocytoma in a pregnant woman presenting with cardiogenic shock from adrenergic myocarditis at 31 weeks’ gestation; hypothesising that gestational hormones may have precipitated catecholamine excess, *in vitro* studies were performed investigating the effect of estradiol and human chorionic gonadotropin (hCG) on epinephrine secretion from cultured cells derived from the patient’s tumor. Finding a striking stimulation of epinephrine by hCG, tumoral expression of LHCGR was then confirmed by RT-PCR and immunohistochemistry. LHGCR expression was confirmed in an additional five of 12 pheochromocytomas, and in silico studies showed pheochromocytomas and paragangliomas have high expression levels of LHCG receptor mRNA relative to 32 solid tumor types of The Cancer Genome Atlas cohort [38]. LHCGR expression was also noted to be higher in PPGLs of Cluster 2 phenotype [38], which are also known to have higher expression of PNMT [39]. These findings are consistent with pregnancy unmasking clinically latent PPGLs in at least some cases via hCG stimulation of tumor growth and epinephrine release.

It is noteworthy similar illicit LHCGR expression has been described in adrenal Cushing’s syndrome in pregnancy [40]; and in aldosterone-producing adenomas presenting in puberty, pregnancy or menopause, associated with somatic *CTNNB1* mutations in combination with *GNA11* or *GNAQ* mutations [41]. It will be interesting to see whether LHCGR expression is similarly elevated in PPGLs presenting in puberty or at menopause.

## Conclusions

Although the outcome of PPGL in pregnancy has improved in recent decades, it remains a potentially lethal diagnosis for mother and baby alike if unrecognised. The key to optimal outcomes is diagnosis of PPGL as the cause of acute onset of paroxysmal symptoms and/or hypertension. Distinguishing PPGL from gestational hypertension or pre-eclampsia remains a challenge which at this stage can only be addressed by clinical education and awareness (Fig. 1). All women with a previous history of PPGL or hereditary PPGL syndrome should have careful preconception assessment to exclude residual or recurrent disease. There should be a low threshold for measuring plasma or urinary metanephrine and normetanephrine in a pregnant woman with hypertension occurring before 20 weeks’ gestation, or acute and/or refractory hypertension particularly if paroxysmal and accompanied by sweating, palpitations and/or headaches. Alpha-blockade should commence once a diagnosis of PPGL is confirmed. An expert team should consider timing of PPGL surgery; many still favour resection if diagnosed before 24 weeks’ gestation, but otherwise surgery should generally be delayed until the post-partum period. Timing and manner of delivery should similarly be considered by an expert team and based upon assessments of fetal wellbeing and control of maternal hypertension; vaginal delivery may be appropriate in selected cases, although Caesarean section is still preferred for many women with larger and/or more biochemically active tumors. All women diagnosed with PPGL in pregnancy should be referred for genetic counselling and testing for hereditary PPGL syndromes.

It is important to note, despite recent outstanding contributions [4, 5] our evidence base for PPGLs in pregnancy consist of case reports and case series with all the attendant selection and publication biases these contain. How can we improve care for women with PPGL in pregnancy? Continuing medical education programs have a role by targeting all healthcare professionals involved in the care of pregnancy; and decision support tools are gaining traction for highlighting the possibility of a rare disease in otherwise common clinical scenarios (e.g. hypertension in pregnancy) [42]. Patient advocacy is also crucial, particularly in the setting of a women with prior history of PPGL or hereditary PPGL syndrome. Without greater awareness of the possibility of PPGL in pregnancy, materno-fetal outcomes are unlikely to improve from current levels.
